# RALF peptides modulate immune response in the moss *Physcomitrium patens*

**DOI:** 10.3389/fpls.2023.1077301

**Published:** 2023-01-27

**Authors:** Anna Mamaeva, Irina Lyapina, Andrey Knyazev, Nina Golub, Timur Mollaev, Elena Chudinova, Sergey Elansky, Vladislav V. Babenko, Vladimir A. Veselovsky, Ksenia M. Klimina, Tatiana Gribova, Daria Kharlampieva, Vassili Lazarev, Igor Fesenko

**Affiliations:** ^1^ Laboratory of System Analysis of Proteins and Peptides, Shemyakin and Ovchinnikov Institute of Bioorganic Chemistry, Russian Academy of Sciences, Moscow, Russia; ^2^ Agrarian and Technological Institute, Peoples Friendship University of Russia (RUDN University), Moscow, Russia; ^3^ Faculty of Biology, Lomonosov Moscow State University, Moscow, Russia; ^4^ Laboratory of Genetic Engineering, Federal Research and Clinical Center of Physical-Chemical Medicine of Federal Medical Biological Agency, Moscow, Russia; ^5^ Department of Molecular and Translational Medicine, Moscow Institute of Physics and Technology (National Research University), Dolgoprudny, Moscow, Russia

**Keywords:** RALF, peptide, stress response, immunity, phytopathogens

## Abstract

**Background:**

RAPID ALKALINIZATION FACTOR (RALFs) are cysteine-rich peptides that regulate multiple physiological processes in plants. This peptide family has considerably expanded during land plant evolution, but the role of ancient RALFs in modulating stress responses is unknown.Results: Here, we used the moss Physcomitrium patens as a model to gain insight into the role of RALF peptides in the coordination of plant growth and stress response in non-vascular plants. The quantitative proteomic analysis revealed concerted downregulation of M6 metalloprotease and some membrane proteins, including those involved in stress response, in PpRALF1, 2 and 3 knockout (KO) lines. The subsequent analysis revealed the role of PpRALF3 in growth regulation under abiotic and biotic stress conditions, implying the importance of RALFs in responding to various adverse conditions in bryophytes. We found that knockout of the PpRALF2 and PpRALF3 genes resulted in increased resistance to bacterial and fungal phytopathogens, Pectobacterium carotovorum and Fusarium solani, suggesting the role of these peptides in negative regulation of the immune response in P. patens. Comparing the transcriptomes of PpRALF3 KO and wild-type plants infected by F. solani showed that the regulation of genes in the phenylpropanoid pathway and those involved in cell wall modification and biogenesis was different in these two genotypes.

**Conclusion:**

Thus, our study sheds light on the function of the previously uncharacterized PpRALF3 peptide and gives a clue to the ancestral functions of RALF peptides in plant stress response.

## 1 Introduction

Plants utilize small secreted peptides as important mediators of many processes, from growth and development to response to stress conditions (Olsson et al., 2019). One of such regulators is the conservative 5 kDa RALF (Rapid Alkalinization Factor) peptide family, which is widely present in terrestrial plants (Campbell and Turner, 2017). The mature RALF peptide contains four cysteine amino acid residues, which form two S-S bonds (Frederick et al., 2019). RALF peptides are cleaved from an inactive protein precursor and S1P protease is shown to be involved in the cleavage of a mature RALF peptide from a nonfunctional precursor at the conserved dibasic RR site (Srivastava et al., 2009; Stegmann et al., 2017).

The tandem duplication is considered to play a dominant role in the evolution of RALFs and this peptide family has expanded considerably during land plant evolution (Cao and Shi, 2012; Campbell and Turner, 2017). For example, 37 members of this family were found in *Arabidopsis thaliana*, 25 in *Arabidopsis halleri*, 20 in *Zea mays*, but only 3 in *Physcomitrium patens* (Campbell and Turner, 2017; Ginanjar et al., 2022). RALF peptides are diverged into four clades based on mature peptide region sequence features (Campbell and Turner, 2017). RALF peptides from I, II and III clades contain a specific protease cleavage site and a conserved YISY motif, which is important for their recognition through receptors (Campbell and Turner, 2017; Xiao et al., 2019). However, RALF peptides from clade IV lack specific cleavage site, in addition, the conserved motif also changes, thus suggesting diverse functions for the representatives from this clade.

Unlike most other plant peptide hormones, RALF peptides bind to membrane-localized receptor-like kinases with a malectin-like extracellular domain instead of a leucine-rich repeat domain (Franck et al., 2018). Receptors of RALF peptides belong to the *Catharanthus roseus* receptor-like kinase (CrRLK1L) family and include FERONIA (FER), CrRLK1Ls ANXUR1 (ANX1), ANX2, and BUDDHA’S PAPER SEAL (BUPS) 1 and BUPS2 (Ge et al., 2017; Ge et al., 2019). In addition, LRE LIKE GPI-AP1 (LLG1) was proposed to function as a coreceptor for FER in AtRALF1 perception in Arabidopsis (Li et al., 2015). In bryophytes, such as *Marchantia polymorpha* L., *P. patens*, and *Sphagnum fallax*, 1, 5 and 7 CrRLK1Ls were identified, respectively (Solis-Miranda et al., 2020). Also, LEUCINE-RICH REPEAT EXTENSINS (LRX) proteins are shown to bind RALF peptides with high affinity (Mecchia et al., 2017; Zhao et al., 2018; Moussu et al., 2020).

The diversity of receptors and co-receptors that can bind different RALF peptides results in their multiple functions and activation of different signaling pathways (Abarca et al., 2021). RALF peptides are involved in the regulation of root growth, pollen tube elongation, the formation of nitrogen-fixing nodules, and inulin accumulation (Murphy and De Smet, 2014; Tena, 2016; Wieghaus et al., 2019). Also, they are involved in the processes of intercellular communication between sporophyte and gametophyte in land plants (Chevalier et al., 2013; Mecchia et al., 2017; Loubert-Hudon et al., 2020). The possible functions of *RALF* genes that have been identified in bryophyte genomes are still being studied. PpRALF1 and PpRALF2 peptides identified in *P. patens* have been shown to promote protonema tip growth and elongation (Ginanjar et al., 2022). Although the function of the PpRALF3 peptide is still unknown. The *Marchantia polymorpha* genome encodes three RALF peptides (Bowman et al., 2017). The single CrRLK1L gene of *Marchantia polymorpha* (MpFER) was recently characterized (Mecchia et al., 2022). Defects in vegetative growth and decreased male fertility characterize plants with reduced MpFER levels. *Mpfer* null mutants and MpFER overexpression lines show dramatic defects in gametophyte cell integrity and morphogenesis (Mecchia et al., 2022).

Besides the regulation of growth and development, RALF peptides are also involved in the regulation of responses to abiotic and biotic stresses (Blackburn et al., 2020). For example, AtRALF8 plays a role in simultaneously controlling responses to drought and nematode attack by allegedly regulating cell wall remodeling (Atkinson et al., 2013). However, as has been shown in Arabidopsis, different RALF peptides may have opposite effects. For example, AtRALF17 increases reactive oxygen species (ROS) production and resistance to *Pseudomonas syringae* pv. tomato, while AtRALF23 has the opposite effect (Stegmann et al., 2017). AtRALF33, a close relative of AtRALF23, was shown to inhibit pathogen elicitor-induced ROS production (Stegmann et al., 2017). In addition, AtRALF23 and AtRALF22 were also shown to participate in regulation of growth and salt stress tolerance by cell wall remodeling (Zhao et al., 2018). The exogenous treatment with synthetic AtRALF1, AtRALF4, AtRALF19 and AtRALF22 reduces elicitor-induced ROS production (Abarca et al., 2021). The corresponding precursors of these AtRALFs contain an S1P cleavage site, but AtRALF6-13, AtRALF15-17, AtRALF20, AtRALF24, AtRALF29-32, AtRALF35-36, which reported to increase elf18-induced ROS production, are not cleaved by this protease (Abarca et al., 2021). Perhaps, the presence of S1P cleavage sites is linked to the negative regulation of the immunity, with some exceptions (Stegmann et al., 2017; Abarca et al., 2021).

The participation of RALF peptides in modulation of the response to biotic stress has been shown not only in Arabidopsis, but also in other plants, including crops (Stegmann et al., 2017; Merino et al., 2019). Generally, RALF peptides are considered negative regulators of the immune response. For example, the RALF–FER complex modulates complex formation between the immune receptor kinase FLAGELLIN-SENSING 2 (FLS2) and its co-receptor BRASSINOSTEROID INSENSITIVE 1-ASSOCIATED RECEPTOR KINASE 1 (BAK1) to inhibit plant immunity (Stegmann et al., 2017; Xiao et al., 2019). Additionally, RALF-induced pH changes may regulate pathogenicity. For instance, the respiratory burst oxidase homologs (RBOHs), that are involved in apoplastic ROS production, have been found to be regulated by FER and related proteins (Franck et al., 2018; Zhang et al., 2020b). FERONIA-like receptor 1 (FLR1) from rice is involved in the regulation of Ca2+ homeostasis in response to rice blast resistance (Luo et al., 2022). However, it is currently unknown how the RALF–CrRLK1L complex regulates ROS production and interferes with calcium signaling during immune response (Liao et al., 2017).

Haruta et al. (2014) demonstrated that the RALF1–FER pathway phosphorylates the proton pump (e.g., AHA2), resulting in transient alkalinization of the extracellular matrix and inhibition of primary root cell elongation (Haruta et al., 2014). Considering that many pathogens, especially fungi, prefer alkaline conditions, the ability of RALFs to alkalize the environment came in very handy (Tena, 2016; Thynne et al., 2017). Many plant pathogens synthesize RALF-like peptides that enhance the development of infection (Tena, 2016; Thynne et al., 2017). F-RALF from *Fusarium oxysporum* f. so. *lycopersici*, which mimics plant RALFs, induces the alkalinization of apoplasts, which activates the orthologous MAPK FMK1 kinase, inhibits root growth and promotes virulence in fungi (Masachis et al., 2016). In tomato and *Nicotiana benthamiana*, a synthetic RALF-like peptide from *F. oxysporum* was also able to induce ROS burst, alkalinization, and activation of MAPKs, as well as inhibit the seedlings growth (Thynne et al., 2017). Moreover, RALF-like peptides from plant root-knot nematodes facilitate the process of infection in Arabidopsis and rice (Zhang et al., 2020a). Although these homologs may have an arguable impact on the potency of pathogen infection (Wood et al., 2020). Alternatively, the plant can suppress pathogen-associated molecular pattern (PAMP)-triggered immunity (PTI) by activating its own *RALFs* expression to shape the root microbiome under phosphate starvation (Tang et al., 2022). However, determining how the RALF–FER pathway modulates immune responses and cell growth by inducing pH changes in a context-dependent manner requires additional research (Zhang et al., 2020b).

The analysis of different plant lineages allows us to gain insight into the possible roles of RALF peptides in the regulation of stress responses (Blackburn et al., 2020). However, the role of ancient RALF peptides in response to stress conditions, particularly phytopathogens, is unknown. *Physcomitrium patens* is a model host plant that is frequently used to study stress response and the evolution of the plant immune system (Ponce de León, 2011; Ponce de León and Montesano, 2013). A diverse range of phytopathogens, such as *Botrytis cinerea*, *Phytophthora infestans, Colletotrichum gloeosporioides*, *Altenaria brassicicola*, *Fusarium* spp. (Akita et al., 2011; Ponce de León, 2011; Lehtonen et al., 2012; Ponce De León et al., 2012; Reboledo et al., 2015; Bressendorff et al., 2016; Overdijk et al., 2016; Marttinen et al., 2020), the bacterium *Pectobacterium carotovorum* (Andersson et al., 2005; Ponce de León et al., 2007), the oomycete *Pythium* (Oliver et al., 2009; Castro et al., 2016) have been shown to infect *P. patens*. Using quantitative proteomic and transcriptomic analysis along with infection with *Pectobacterium carotovorum* and *Fusarium solani*, we studied the role of three PpRALF peptides from the model bryophyte *Physcomitrium patens* in the immune stress response. Our study showed the specific role of PpRALF3 in negative regulation of plant stress response.

## 2 Materials and methods

### 2.1 Plant growth conditions and treatments

*Physcomitrella patens* subsp. *patens* (*Physcomitrium patens* “Gransden 2004”, Freiburg) protonemata were grown on BCD medium supplemented with 5 mM ammonium tartrate (BCDAT) and/or 0.5% glucose with 1.5% agar (Helicon, Moscow, Russian Federation) in a Sanyo Plant Growth Incubator MLR-352H (Panasonic, Osaka, Japan) with a photon flux of 61 μM/m^2^•s during a 16-hour photoperiod at 24°C in 9 cm Petri dishes (Nishiyama et al., 2000). For proteomic and qRT-PCR analyses, the protonemata were grown in liquid BCDAT medium and collected on day 7. The gametophores were grown on free-ammonium tartrate BCD medium under the same conditions, and 8-week-old gametophores were used for analysis. For analysis of abiotic stress resistance, protonemata were grown on BCD medium without ammonium tartrate supplemented with 150 mM NaCl or 2 μM paraquat (PQ). For *PpRALFs* expression analysis, 7-day-old protonemata were treated with 100 mM hydrogen peroxide for 2 hours.

For morphological analysis, protonema tissue 2 mm in diameter was planted on 9 cm Petri dishes on BCD and BCDAT media.

For growth rate measurements, photographs were taken at day 30 of subcultivation. Protonemal tissues and cells were photographed using a Microscope Digital Eyepiece DCM-510 attached to a Stemi 305 stereomicroscope (Zeiss, Germany).

### 2.2 Generation of knockout mutant lines

*PpRALF1* (Pp3c3_15280V3), *PpRALF2* (Pp3c6_7200V3) and *PpRALF3* (Pp3c25_4180V3) knockout lines were created using the CRISPR/Cas9 system (Collonnier et al., 2017). The coding sequences were used to search for guide sequences preceded by a *Streptococcus pyogenes* Cas9 PAM motif (NGG) using the web tool CRISPOR (http://crispor.tefor.net/). The guide sequence closest to the translation start site (ATG) was selected for cloning (**Supplementary Table S1** and **Figure S1**). These sequences were cloned into plasmid pBB (Fesenko et al., 2019), yielding the final complete sgRNA expression cassette. Protoplasts were obtained from protonemata as described previously (Fesenko et al., 2015) and transformed by the PEG transformation protocol (Schaefer and Zrÿd, 1997) using a mixture of three plasmids 1) one of the pBB plasmid carrying guide RNA expression cassette; 2) pACT-CAS9 carrying the CAS9 gene; 3) pBNRF plasmid carrying resistance gene to G418. The plasmids pACT-CAS9 and pBNRF were kindly provided by Dr. Fabien Nogué. Independent knockout mutant lines have been obtained. Double knockout lines were obtained by simultaneous transformation of two plasmids with the corresponding guide RNAs.

### 2.3 DNA and RNA isolation and quantitative reverse transcription PCR

Genomic DNA from gametophores was isolated using a commercial kit (Biolabmix, Russia), according to the manufacturer’s recommendations. Total RNA from gametophores and protonemata was isolated using TRIzol™ Reagent according to the manufacturer’s recommendations. RNA quality and quantity were evaluated using electrophoresis on agarose gel with SYBR Green (Biolabmix, Russia). Total RNA concentration of samples was precisely measured using a Nanodrop™One (Thermo Fisher Scientific, USA).

cDNA was synthesized using the MMLV RT kit (Evrogen, Russian) according to the manufacturer’s recommendations. OligodT primers were used to prepare cDNA from 2 µg total RNA after DNase treatment.

Real-time PCR was performed using the HS-qPCR SYBR Blue (2x) (Biolabmix, Russia) on a LightCycler^®^96 (Roche, Germany). Three biological and three technical replicates were used for the qPCR. Primers for the target genes could be found in **Supplementary Table S1**.

For qPCR analysis of infection severity, primers were designed for *F. solani* or selected from previous research (Kabir et al., 2020) for *P. carotovorum* to specifically amplify pathogenic DNA from the infected moss plants (**Supplementary Table S1**).

### 2.4 Pathogen inoculation and treatments

Phytopathogens *Pectobacterium carotovorum subsp. atrosepticum* (strain ECPA16 NCBI № OL677456) and *Fusarium solani* (20 МККК1 NCBI № OQ073458) were used to infect moss plants. Bacterial culture of *P. carotovorum* was grown for 18 hours, after which the optical density of the bacterial suspension was measured on a spectrophotometer at 600 nm. Based on preliminary results, a concentration of ~10^7^ cfu/mL was chosen for the further experiments. To obtain this concentration the initial bacterial suspension was diluted 1000 times with sterile water. Therefore, sterile water-treated plants were used as a control. For plant treatment, 15 µl of prepared overnight culture with a final concentration 9.5×10^6^ cfu/mL were used. After each experiment, a diluted bacterial suspension was grown on medium for subsequent CFU counts. *F. solani* spores were obtained from a solid medium culture and suspension with final concentration 8.3×10^5^ spores/mL was applied. *Fusarium solani* conidia were also diluted with sterile water, and the concentration of conidia was counted under a microscope. Petri dishes with infected plants were cultivated at room temperature under standard conditions (16 hours day/8 hours night). Infected plants were collected after 7 days and frozen in liquid nitrogen.

### 2.5 Protein extraction and trypsin digestion

The three independent biological repeats for each genotype were used for comparative proteomic analysis. Protein extraction and trypsin digestion we conducted as described previously (Faurobert et al., 2007; Fesenko et al., 2021b). iTRAQ labeling (Applied Biosystems, Foster City, CA, USA) was conducted according to the manufacturer’s manual. Proteins were labeled with the iTRAQ tags as follows: wild-type biological replicates – 113, 114, 115 isobaric tags; *PpRALF1*, *PpRALF2* and *PpRALF3* KO biological replicates – 116, 117, 118 isobaric tags for each mutant line.

### 2.6 LC-MC/MC analysis and protein identification and quantification

The LC–MS/MS analysis was performed as described earlier (Fesenko et al., 2021a). Tandem mass spectra were searched with PEAKS Studio version 8.0 software (Bioinfor Inc., CA, USA) against a custom database containing 32 926 proteins from annotated genes in the latest version of the moss genome v3.3 (Lang et al., 2018), 85 chloroplast proteins, and 42 mitochondrial proteins. The search parameters were the following: a fragmentation mass tolerance of 0.05 Da; parent ion tolerance of 10 ppm; fixed modification – carbamidomethylation; variable modifications - oxidation (M), deamidation (NQ), and acetylation (protein N-term). The results were filtered by a 1% FDR, but with a significance threshold of not less than 20 (equivalent to a P-value of less than 0.01). The results were filtered by a 1% false discovery rate (FDR). PEAKS Q was used for iTRAQ quantification. Normalization was performed by averaging the abundance of all peptides. The median values were used for averaging. Although iTRAQ quantification usually underestimates the amount of real fold changes between two samples (Ow et al., 2009), we used a very strict filter for differentially expressed proteins. The threshold for calling a protein differentially abundant was calculated based on an s0 parameter (s0 = 0.1) as described previously (Schessner et al., 2022) with a permutation test repeated 100 times and Benjamini and Hochberg FDR correction (FDR_BH < 1%).

### 2.7 RNA-seq analysis

DNase treatment was carried out with TURBO DNA-free kit (Thermo Fisher Scientific, Waltham, MA, USA), in volumes of 50 µl. RNA cleanup was performed with the Agencourt RNA Clean XP kit (Beckman Coulter, Brea, USA). The concentration and quality of the total RNA were checked by the Quant-it RiboGreen RNA assay (Thermo Fisher Scientific) and the RNA 6000 Pico chip (Agilent Technologies, Santa Clara, CA, USA), respectively.

RNA libraries were prepared using NEBNext Poly(A) mRNA Magnetic Isolation Module and the NEBNext Ultra II Directional RNA Library Prep Kit (NEB), according to the manufacturer’s protocol. The library underwent a final cleanup using the Agencourt AMPure XP system (Beckman Coulter) after which the libraries’ size distribution and quality were assessed using a high sensitivity DNA chip (Agilent Technologies). Libraries were subsequently quantified by Quant-iT DNA Assay Kit, High Sensitivity (Thermo Fisher Scientific). Finally, equimolar quantities of all libraries (10 pM) were sequenced by a high throughput run on the Illumina HiSeq 2500 using 2 × 100 bp paired-end reads and a 1% Phix spike-in control.

The adaptors and low-quality sequences were removed from raw reads by Trimmomatic v0.39 (Bolger et al., 2014). Clean reads were aligned to the *P. patens* v3.3 reference genome (download from the website: https://phytozome-next.jgi.doe.gov) using HISAT2 v2.1.0 (Kim et al., 2015) and the alignments were sorted with Samtools (Li et al., 2009). The expression abundances of mapped reads were counted by the FeatureCounts tool (Liao et al., 2014). Differential expression analysis was performed by EdgeR package (Robinson et al., 2010). The genes were defined as differentially expressed genes (DEGs) with adjusted p-value ≤ 0.05 and fold change ≥1.0.

### 2.8 Cell wall staining and ROS detection

Protoplasts were prepared from protonemata as described previously (Fesenko et al., 2015) and incubated for 48 hours at solid BCD agar medium. Regenerated protoplasts were stained with 10 µg/ml Calcofluor White (Fluorescent Brightener 28) for 5 minutes. After that, protoplasts were analyzed using fluorescent microscope (Axio Imager M2, Zeiss, Germany) at λex = 365 nm, BS FT 395, and λem = 445nm/50 nm (Filter set 49 DAPI, Zeiss, Germany).

The fluorescent dye 2,7-Dichlorofluorescin Diacetate (DCFH-DA, Sigma-Aldrich, USA) was used to identify intracellular ROS. Using a spatula, seven-day-old protonema filaments were removed from the agar surface and transferred to mQ. Protonemata were then treated with 0.0025% driselase (diluted in mQ) for 1 minute or mQ water as a control and incubated with 10 µM DCFH-DA for 15 minutes in total. The No. 44 filter (λex BP 475 nm/40 nm; λem BP 530 nm/50 nm) was used for DCFH-DA fluorescence detection on the fluorescent microscope Axio Imager M2 (Zeiss) with an AxioCam 506 mono digital camera. Data on the fluorescence intensity were obtained from the related Zeiss software Zen.

### 2.9 Bioinformatic analysis

Protein–protein interaction networks were constructed using STRING v.10 (www.string-db.org) with the default options (Szklarczyk et al., 2014). The visualization of the protein interaction was performed with Cytoscape software (Shannon et al., 2003). The GO enrichment analysis was conducted by g:Profiler (Raudvere et al., 2019). Multiple alignments were created using the MAFFT algorithm (Katoh et al., 2019) and visualized using Jalview software (Waterhouse et al., 2009). IQ-TREE multicore version 2.2.0 (Nguyen et al., 2015) was used to conduct a maximum likelihood (ML) analysis with 1000 ultrafast bootstrap replicates (Minh et al., 2013). The model FLU+F+G4 was chosen as the best-fit model by the in-built ModelFinder program (Kalyaanamoorthy et al., 2017) according to the Bayesian Information Criterion (BIC). Principal Component Analysis (PCA) was performed using the iFeature tool (Chen et al., 2018).

### 2.10 Statistics

Statistical analysis and visualization were made in Python v. 3.7.5 (Van Rossum and Drake, 1995) using modules scipy 1.5.2 (Virtanen et al., 2020), seaborn 0.11.1 (Waskom, 2021), numpy 1.20.1, pandas 1.2.3 (McKinney, 2012). For two- or more-way analysis of variance (ANOVA), Tukey’s honestly significant difference (HSD) tests based on multiple comparisons of means were applied to determine which pairwise comparisons were statistically significant. Differences were considered to be significant at p < 0.05.

## 3 Results

### 3.1 The amino acid composition of PpRALFs

**Figure 1:**
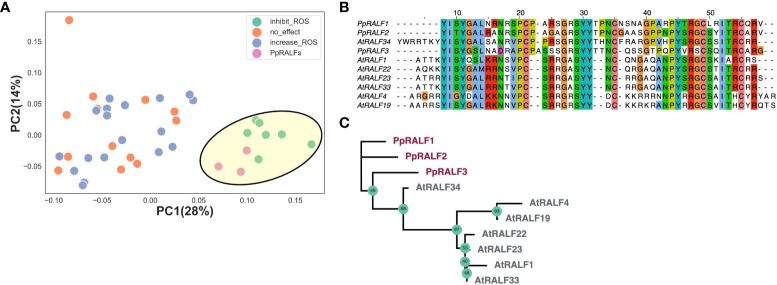
**(A)** Principal Component Analysis (PCA) of amino acid composition, calculated by iFeature tool (Chen et al., 2018) for AtRALF and PpRALF peptides. Moss PpRALFs were clustered with reported immune-related RALF peptides from Arabidopsis, such as AtRALF1, AtRALF4, AtRALF19, AtRALF22, AtRALF23, AtRALF33, and AtRALF34. **(B)** The multiple pairwise alignment of PpRALFs and immune-related AtRALFs (AtRALF1, AtRALF4, AtRALF19, AtRALF22, AtRALF23, AtRALF33, AtRALF34). **(C)** An unrooted phylogenetic tree with RALF amino acid sequences from *Physcomitrium patens* (PpRALF1, PpRALF2, PpRALF3) and immune-related AtRALFs (AtRALF1, AtRALF4, AtRALF19, AtRALF22, AtRALF23, AtRALF33, AtRALF34); Ultra-fast bootstrap (1000 replicates) support values are shown.

The members of a RALF peptide family possess functional heterogeneity in vascular plants (Blackburn et al., 2020), including the role in modulation of immune response. In Arabidopsis, exogenous application of synthetic AtRALF23, AtRALF33, AtRALF34 are shown to inhibit production of reactive oxygen species (ROS) induced upon treatment with immune elicitors (such as elf18) and inhibit seedling and root growth, whereas AtRALF17, AtRALF24, AtRALF32 and some others were able to induce ROS production (Abarca et al., 2021). The previous phylogenetic analysis divided the RALFs into four clades, and peptide sequences from different clades could be distinguished based on analysis of their physico-chemical properties (Campbell and Turner, 2017). To test how the overall amino acid composition of RALF peptides is related to their possible functions, we used Principal Component Analysis (PCA). The results of PCA analysis showed a distinct pattern in which AtRALF peptides that inhibit the production of pathogen-induced ROS (Abarca et al., 2021) were clustered together. We next found that PpRALFs were clustered with AtRALFs that inhibit elicitor-induced ROS production (**Figure 1A**; **Supplementary Table S2**). This result is consistent with previous phylogenetic analysis (Ginanjar et al., 2022), where PpRALFs were grouped with AtRALF22, AtRALF23, AtRALF33, and AtRALF34. These findings suggest that the overall amino acid composition of RALF peptides, to some extent, reflects their functional biases, and PpRALFs are related to AtRALFs that negatively regulate immune response.

In *Physcomitrium patens*, PpRALF1 and PpRALF2, which are members of Clade III (Campbell and Turner, 2017), have shown to promote protonema tip growth and elongation (Ginanjar et al., 2022). PpRALF3, the third member of the RALF peptide family in P. patens, contains a substitution in the conserved “RGC” motif (**Figure 1B**). Our phylogenetic analysis revealed that PpRALF3 diverged from PpRALF1 and PpRALF2 and formed a group with AtRALF peptides (**Figure 1C**). The AtRALFs that were clustered together with PpRALF peptides (**Figure 1A**) belong to Clades 1, 2 and 3 (Campbell and Turner, 2017), implying the absence of the correlation between RALF functions in immune response and division into clades in this case. However, the role of PpRALFs in responding to stress conditions is unknown.

### 3.2 The knockout of *PpRALF*s resulted in changes of cell wall-associated proteins

PpRALF1 and PpRALF2 peptides are previously shown to promote tip growth and elongation of protonemata filaments in *P. patens*, but the functions of PpRALF3 peptide are currently unknown (Ginanjar et al., 2022). The chemical synthesis of RALF peptides, as well as the generation of recombinant peptides, is associated with certain difficulties. Proper bonding and folding of peptides require the right conditions, otherwise peptides may not work the way they do, or a higher concentration could be required (Abarca et al., 2021). Since this may not reflect the actual effect of the peptides, we decided to use only knockout lines. At least two independent knockout lines for each *PpRALF* gene and double knockout lines - *PpRALF1* and *PpRALF3*; *PpRALF2* and *PpRALF3* were generated by CRISPR/Cas9 technology (**Table 1**; **Supplementary Figure S1**). However, we failed to obtain triple and double *PpRALF1* and *PpRALF2* knockout lines after several attempts. Probably, this is due to their important roles in regulation of moss growth and development.

**Table 1:** The list of obtained mutant lines.

Phytozome ID	Name	The number of mutant lines
Pp3c3_15280	*PpRALF1 KO*	2
Pp3c6_7200	*PpRALF2 KO*	2
Pp3c25_4180	*PpRALF3 KO*	2
Pp3c3_15280, Pp3c25_4180	*PpRALF1,3 KO*	1
Pp3c6_7200, Pp3c25_4180	*PpRALF2,3 KO*	1

**Figure 2:**
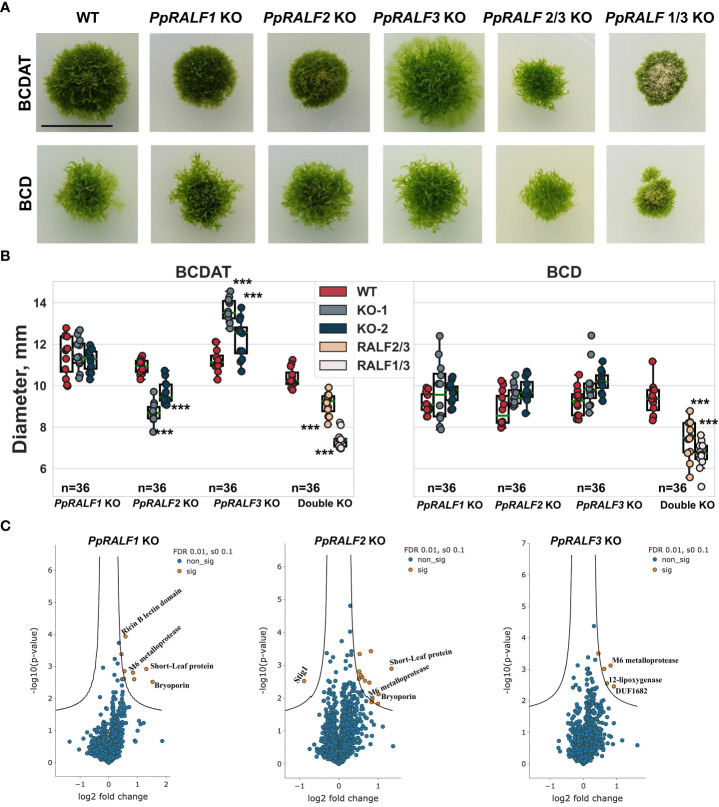
**(A)** Phenotype of 30-d-old plants of wild-type and the obtained knockout lines grown on two mediums with and without ammonium tartrate. Scale bar: 10 mm; **(B)** Wild-type and knockout lines’ plant diameters when grown on media containing (BCDAT) and without ammonium tartrate (BCD). For each experiment, the diameters of the wild-type and knockout plants n = 12 for each genotype) are displayed. Analysis of variance and Tukey’s HSD *post hoc* tests were performed ***P < 0.001 (compared to wild-type plants); **(C)** Volcano plots of the entire set of proteins quantified during iTRAQ analysis of *PpRALF1*, *PpRALF2* and *PpRALF3* KO lines, respectively. Proteins significantly changed in abundance are depicted in orange; Pp3c15_830 M6 family metalloprotease domain-containing protein, Pp3c14_22870 SHORT-LEAF protein, Pp3c26_120 bryoporin, Pp3c15_13040 12-lipoxygenase, Pp3c16_14730 Stigma-specific protein, Stig1, Pp3c5_6620 DUF1682, Pp3c3_10250 Ricin B, lectin-containing protein.

It has been previously shown that knockout of *PpRALF* genes results in reducing the formation of caulonema filaments (Ginanjar et al., 2022). Therefore, we tested the growth of our mutants on medium with and without ammonium tartrate (BCDAT/BCD; **Figure 2A**). The ammonium tartrate reduces the transition into caulonemal cells and promotes chloronemal branching (Vidali and Bezanilla, 2012). Similar to the previous study (Ginanjar et al., 2022), we observed the reduced formation of caulonema filaments in mutant genotypes (**Supplementary Figure S2**). The morphology of wild-type and single knockout mutants was similar, but the diameter of *PpRALF3* KO plants was significantly larger than wild-types on the BCDAT medium (**Figures 2A, B**; ANOVA with post-hoc Tukey HSD *P* < 0.001). In contrast, the growth rate of *PpRALF1* KO lines did not differ from wild-type on all media examined (**Figures 2A, B**). In addition, *PpRALF2* KO plants were significantly smaller than wild-type plants on the medium with ammonium tartrate (**Figure 2B**, ANOVA with post-hoc Tukey HSD *P* < 0.001). Both double knockouts showed significant inhibition of the growth rate on medium with and without ammonium tartrate (**Figures 2A, B**; ANOVA with post-hoc Tukey HSD *P* < 0.001). *PpRALF1,3* KO plants differed from other knockout plants, and cell death was observed on both mediums.

In addition, the gametophores were significantly longer in both *PpRALF3* KO lines in comparison to other genotypes (**Supplementary Figure S3**; ANOVA with post-hoc Tukey HSD *P* < 0.001). Thus, our results are in line with previously obtained data on morphology and phenotypes of *PpRALF* knockouts (Ginanjar et al., 2022). In addition, our results suggest that all PpRALFs are functional and might play different roles, beyond the regulation of only growth processes.

Using isobaric tags for relative and absolute quantification (iTRAQ), we further compared the proteomes of *PpRALF1*, *PpRALF2*, and *PpRALF3* knockout lines and wild-type plants. In total, we identified 2723 protein groups in *PpRALF1* KO, 3074 protein groups in *PpRALF2* KO, and 3078 protein groups in *PpRALF3* KO (**Supplementary Table S3**). Although iTRAQ quantification usually underestimates the amount of real fold changes between two samples (Ow et al., 2009), we used a very strict cut-off to reliably identify differentially abundant protein groups (DAPs), such as an s0 parameter equal to 0.1 and multiple hypothesis correction that was performed based on a permutation test (see Methods). In contrast to the transcriptomes of the *PpRALF* knockout lines that have already been published (Ginanjar et al., 2022), we have not found any major differences between the knockout and wild-type proteomes. This suggests that PpRALF peptides have mostly specific regulatory roles. In *PpRALF1* KO plants only 8 protein groups were significantly changed (FDR BH < 1%, s0 = 0.1; **Figure 2C**; **Supplementary Table S3**) in comparison to wild-type plants and all of these DAPs were downregulated. The knockout of *PpRALF2* gene resulted in downregulation of 15 protein groups (FDR_BH < 1%, s0 = 0.1; **Figure 2C**; **Supplementary Table S3**) and upregulation of an uncharacterized 257 aa protein Pp3c16_14730, containing predicted signal peptide. In *PpRALF3* KO plants only 5 protein groups were significantly downregulated (FDR_BH < 1%, s0 = 0.1; **Figure 2C**; **Supplementary Table S3**).

We further compared these DAPs from all knockout lines and found a core set of four proteins that were changed in at least two single-gene knockout lines. For example, we revealed that a protein Pp3c15_830 (M6 family metalloprotease domain-containing protein) was downregulated in *PpRALF1*, *PpRALF2* and *PpRALF3* KO lines. However, the role of such proteins in RALF signaling has not been previously described. Another DA protein that belonged to M6 metalloproteases - Pp3c13_22390 was downregulated only in the *PpRALF1* KO line. We also identified two proteins that were downregulated in both *PpRALF1* and *PpRALF2* KO lines. One of them - Pp3c26_120 (Bryoporin) is known to be important for a response to osmotic stress in mosses (Hoang et al., 2009). The other protein, Pp3c14_22870 (SHORT-LEAF), is encoded by a bryophyte-specific gene that represents a family of near-perfect tandem direct repeat (TDR)-containing proteins and has been shown to regulate gametophore development in moss (Mohanasundaram et al., 2021). We also identified a LOX3 protein (Pp3c15_13040) that was downregulated in *PpRALF2* and *PpRALF3* KO lines, which might be involved in the arachidonic acid metabolism, suggesting the possible role of these paralogs in biotic stress response.

Thus, the knockout of *PpRALF* genes resulted in downregulation of some proteases and previously uncharacterized membrane proteins. To determine whether these changes in proteomes of mutant lines affected the cell wall regeneration processes, we explored the process of protoplast regeneration in the knockout and wild-type genotypes. The protoplasts were dyed with Calcofluor White fluorescent dye that is widely used to visualize cellulose, callose, and other β-glucans in the plant cell wall (Maksimov et al., 2016; Herrera-Ubaldo and de Folter, 2018). On average, the cell wall regenerated 80% faster in *PpRALF2* KO lines (chi-square *P* < 0.001) and 46% faster in *PpRALF3* KO lines (chi-square *P* < 0.001) than in wild-type cells after two days of regeneration process (**Supplementary Figure S4**). We found no significant differences in the cell wall regeneration rate between *PpRALF1* KO-1 and wild-type plants.

### 3.3 The *PpRALF3* knockout lines are more tolerant to abiotic stress factors

Some members of the RALF peptide family are shown to modulate the abiotic stress response in angiosperms (Zhao et al., 2018). Therefore, we next sought to expand our understanding of PpRALFs functions under abiotic stress conditions. At first, we used previously obtained RNA-seq data (Khraiwesh et al., 2015) to analyze the expression of *PpRALF* genes under salt, drought, and cold treatments. According to this study, *PpRALF1* was significantly upregulated in all stress conditions after 0.5 hours and under cold treatment after 4h but downregulated under drought and salt treatment after 4 hours. In addition, the *PpRALF2* gene was significantly upregulated at drought after 0.5 hours and at cold after 4 hours as well. The *PpRALF3* transcripts were detected only under salt and drought treatment, suggesting its possible role in stress response.

**Figure 3:**
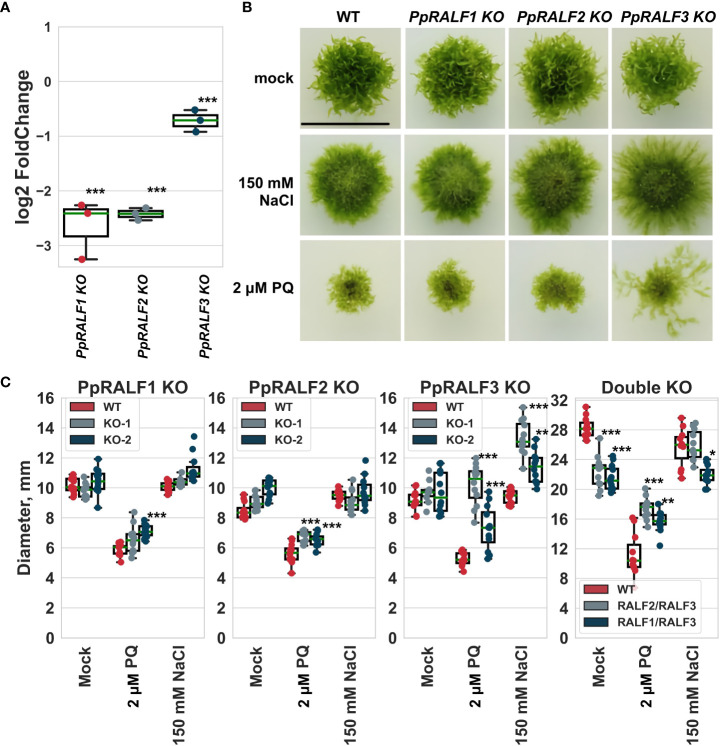
**(A)** The results of quantitative reverse transcription PCR (RT-qPCR) analysis of the transcriptional level of *PpRALFs* under hydrogen peroxide treatment for 2 hours. Analysis of variance and Tukey’s HSD *post hoc* tests were performed ***P < 0.001. Log2FoldChanges represent the logarithmic fold change of the PpRALF genes transcription in treated samples relative to control samples, normalized to a reference gene (2-ΔΔCT values); **(B)** Phenotype of 30-d-old plants of wild-type and the knockout lines grown on medium supplemented with 150 mM NaCl or 2 μM paraquat. Scale bar: 10 mm; **(C)** The measured diameter of moss plants grown on medium supplemented with 150 mM NaCl or 2 μM paraquat. Analysis of variance and Tukey’s HSD *post hoc* tests were performed ***P < 0.001, ** P<0.01, * *P*<0.05 (comparison to the corresponding wild-type plants).

We also assessed the transcriptional level of all *PpRALFs* under hydrogen peroxide treatment for 2 hours. According to qRT-PCR, all *PpRALFs* were significantly downregulated under this treatment (**Figure 3A**; ANOVA with post-hoc Tukey HSD *P* < 0.001). However, the transcriptional level of *PpRALF3* decreased less than that of other *PpRALFs.*

Taking into account the role of RALF peptides, such as AtRALF22/23, in regulation of salt stress tolerance in Arabidopsis (Zhao et al., 2018), we further assessed the growth rate of *PpRALF* KO plants upon the oxidative (2 μM paraquat) and salt (150 mM NaCl) treatments. We have chosen the salt concentration of 150 mM NaCl for these experiments based on our assessment of wild-type plant phenotypes at different salt concentrations (**Supplementary Figure S5**). Our experiments showed that wild-type and knockout plants were affected by 150 mM NaCl, confirming the adverse effect of high salt concentration on moss growth and development (**Figure 3B**). Herewith, the diameters of wild-type and *PpRALF1* and *2* KO plants were similar under salt treatment, except for *PpRALF3* KO lines. The diameter of *PpRALF3* KO lines was significantly larger (ANOVA with post-hoc Tukey HSD *P* < 0.001) under salt treatments compared to mock-treated and wild-type plants due to diffusion of protonemal filaments (**Figures 3B, C**). Under salt treatment, there were some differences in the diameters of the *PpRALF3* KO-1 and KO-2 lines, but the protonemal filaments of both knockouts tended to be longer than those of wild-type plants. The plant size of double knockouts was unaffected by 150 mM NaCl, and only the diameter of *PpRALF1,3* KO was slightly, albeit significantly smaller than that of the wild-type (**Figure 3C**).

Paraquat is a commonly used herbicide that significantly increases the production of reactive oxygen species (ROS) and inhibits the regeneration of reducing equivalents and compounds required for the antioxidant system’s activity (Lascano et al., 2012). Under the oxidative stress conditions induced by 2 μM paraquat, both wild-type and mutant plants were severely affected (**Figure 3B**). Despite the similarly affected phenotypes, the diameter of *PpRALF2* KO plants was slightly, albeit significantly, larger (**Figures 3B, C**; ANOVA with post-hoc Tukey HSD *P* < 0.001). *PpRALF3* KO-1 and KO-2 lines exhibited a phenotype characterized by protonemal segments that were much longer than in wild-type plants and other knockouts (**Figure 3B**). Because of this, the diameter of *PpRALF3* KO plants was significantly larger than that of other genotypes (**Figure 3C**; ANOVA with post-hoc Tukey HSD *P* < 0.001). In addition, both double KO mutant lines were less sensitive to paraquat in comparison to wild-type plants (**Figure 3C**). Thus, our results showed that *PpRALF3* is responsive to adverse conditions, and its knockout lines are more tolerant to growth inhibition during an abiotic stress response.

As has been shown for angiosperm RALFs, these results point to the role of PpRALF3 in the response to abiotic stress factors. Previously it has been shown that the overexpression of AtRALF22 or AtRALF23 resulted in increased sensitivity to salt stress (Zhao et al., 2018). However, further research will be required on this topic.

### 3.4 The knockout of *PpRALF2* and *PpRALF3* increases resistance to phytopathogens

It has been previously shown that some RALF peptides can modulate immune response in vascular plants (Stegmann et al., 2017; Zhang et al., 2020b; Abarca et al., 2021). For example, treatment with AtRALF peptides increased or reduced ROS production under induction of immune response by elf18 (Abarca et al., 2021). To determine whether the knockout of *PpRALF* genes interfere with immune response in bryophytes, we next analyzed ROS production in *P. patens* wild-type and knockout lines under driselase treatment. Driselase is a mix of cell wall–degrading enzymes from *Basidiomycetes* sp. and this enzyme mix is comparable to the enzyme cocktail released by fungal pathogens during infection (Engelsdorf et al., 2018).

**Figure 4:**
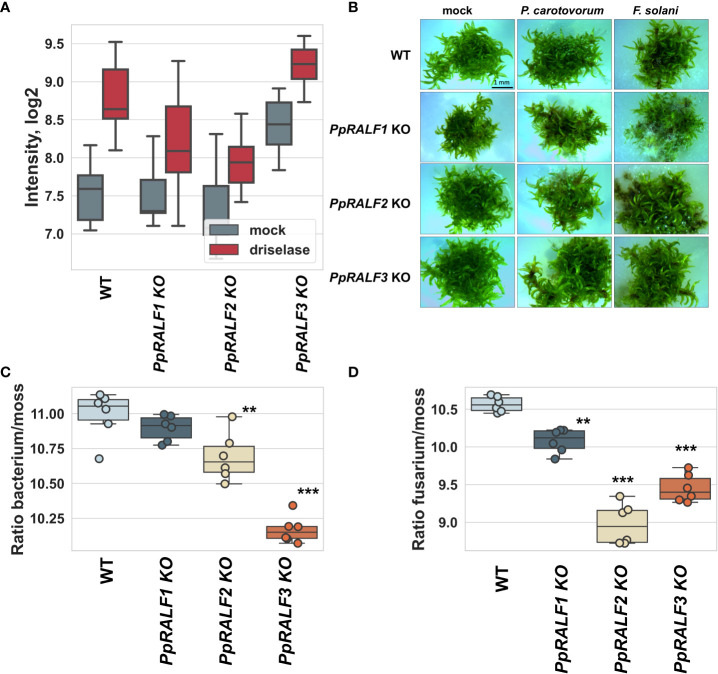
**(A)** Effect of *PpRALF* gene knockouts on ROS production in wild-type and knockout lines treated with 0.0025% driselase; **(B)** Symptom development at 7 dpi in wild-type, *PpRALF1*, *PpRALF2* and *PpRALF3* KO moss plants. Scale bar: 1 mm; **(C)** Ratios of *P. carotovorum* DNA levels to *P. patens* genomic DNA that were estimated by qPCR analysis. **(D)** Ratios of *F. solani* DNA levels to *P. patens* genomic DNA that were estimated by qPCR analysis. The results of six independent experiments are shown. Analysis of variance and Tukey’s HSD *post hoc* tests were performed for qPCR data. **P < 0.01, ***P < 0.001.

We found that ROS production was significantly reduced in *PpRALF1* and *PpRALF2* KOs in comparison to wild-type plants and *PpRALF3* knockout line after driselase treatment (**Figure 4A**; ANOVA with post-hoc Tukey HSD *P* < 0.001). Moreover, the background level of ROS production in the *PpRALF3* knockout line without treatment was significantly higher than in the other genotypes (**Figure 4A**; ANOVA with post-hoc Tukey HSD *P* < 0.001). Background ROS production was also increased in the second *PpRALF3* knockout line relative to wild-type plants (**Supplementary Figure S6A**). Additionally, we analyzed ROS production in double knockout lines following driselase treatment. We found that the background level of ROS production in the *PpRALF* 1,3 KO line was significantly higher than in the wild-type and *PpRALF* 2,3 knockout lines (ANOVA with post-hoc Tukey HSD *P* < 0.001; **Supplementary Figure S6B**). Double knockouts and wild-type plants did not differ in their ROS elevation in response to driselase treatment. Based on these results, we suggest that PpRLAFs modulate the immune response of *P. patens*.

To test whether PpRALFs contribute to the *P. patens* defense response, we used two well-known phytopathogens: *Pectobacterium carotovorum subsp. atrosepticum* and *Fusarium solani*. *P. carotovorum* has been previously shown to infect *P. patens* (Andersson et al., 2005; Ponce de León et al., 2007). The phytopathogenic fungus with a wide range of hosts, *Botrytis cinerea*, is commonly used for the study of immune response in bryophytes (Ponce De León et al., 2012; Reboledo et al., 2021). However, *B. cinerea* is aggressive, and moss plants rapidly deteriorated after inoculation (Ponce De León et al., 2012; Reboledo et al., 2021). Therefore, to compare the immune responses of different genotypes, we selected *Fusarium solani*, which caused only mild symptoms (**Figure 4B**). These pathogens also show distinct symptoms of infection, such as brown spots caused by tissue necrosis (Akita et al., 2011; Alvarez et al., 2016). To test whether the colonization and growth of *P. carotovorum* and *F. solani* differ between wild-type and knockout lines, moss plants were collected at 7 days post inoculation (dpi), and the ratio of pathogen DNA to plant DNA concentrations was measured using quantitative PCR analysis as was described previously (Castro et al., 2016).

According to our experiments, *P. carotovorum* growth rate was significantly higher in wild-type plants compared to *PpRALF2* KO and *PpRALF3* KO mutant lines (**Figure 4C**; ANOVA with post-hoc Tukey HSD *P* < 0.001). However, the difference in *P. carotovorum* growth rate between wild-type and *PpRALF1* KO plants was not significant (**Figure 4C**). The growth of *F. solani* was significantly reduced in all *PpRALF* knockouts in comparison to wild-type plants (**Figure 4D**; ANOVA with post-hoc Tukey HSD *P* < 0.001). *PpRALF2* KO and *PpRALF3* KO mutant lines were found to be the most resistant to *F. solani* infection. These findings suggested that knocking out the *PpRALF* genes increased *P. patens* resistance to phytopathogens. Apparently, PpRALF2 and PpRALF3 play a key role in negative regulation of immune response in *P. patens*, as was shown for some members of the RALF family in Arabidopsis.

### 3.5 Transcriptome response during *F. solani* infection in *PpRALF3* KO line

**Figure 5:**
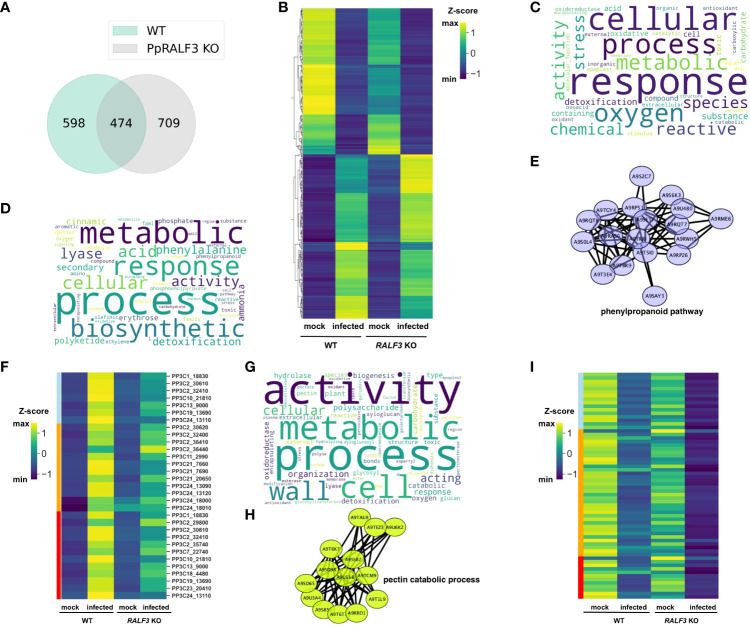
**(A)** Venn diagram depicting overlapping differentially regulated genes (DEGs) between wild-type and *PpRALF3* KO lines; **(B)** A heat map depicting transcript levels of overlapping between wild-type and *PpRALF3* KO differentially expressed genes. Normalized transcripts per million (TPM) values are shown; **(C)** Gene ontology (GO) terms bias word cloud. Word cloud of enriched GO terms for overlapped DEGs between wild-type and *PpRALF3* KO lines is shown (false discovery rate (FDR) adjusted *P*-values ≤ 0.05); **(D)** GO terms bias word cloud. Word cloud of enriched GO terms in wild-type plants is shown (false discovery rate (FDR) adjusted *P*-values ≤ 0.05); **(E)** Protein–protein interaction networks of a group of upregulated differentially expressed genes in wild-type plants that belong to phenylpropanoid metabolic process. Genes are indicated with nodes, and interactions between proteins are represented by edges; **(F)** A heat map depicting transcript levels of DEGs that belong to “phenylpropanoid biosynthetic process” (lightblue), “​​polyketide metabolic process” (orange), “aromatic amino acid family metabolic process” (red). Normalized transcripts per million (TPM) values are shown; **(G)** GO terms bias word cloud. Word cloud of enriched GO terms in *PpRALF3* KO plants is shown (false discovery rate (FDR) adjusted *P*-values ≤ 0.05); **(H)** Protein–protein interaction networks of a group of down regulated differentially expressed genes in *PpRALF3* KO plants that belong to the pectin catabolic process. Genes are indicated with nodes, and interactions between proteins are represented by edges. **(I)** A heat map depicting transcript levels of DEGs that belong to “pectin metabolic process” (lightblue), “​​cell wall organization or biogenesis” (orange), “xyloglucan:xyloglucosyl transferase activity” (red). Normalized transcripts per million (TPM) values are shown.

We found that both *P. carotovorum* and *F. solani* propagated much more slowly in *PpRALF2* and 3 KO genotypes than in wild-type plants and *PpRALF1* knockout lines. This suggests that these knockout genotypes are more tolerant to phytopathogens. To expand our understanding of the roles of PpRALFs in immune stress responses, we then used RNA sequencing (RNA-seq) analysis to compare the transcriptome responses of *PpRALF3* KO and wild-type plants during *F. solani* infection. Twelve paired-end RNA-Seq libraries were generated from three biological replicates of mock-inoculated and *F. solani*-infected wild-type and *PpRALF3* KO plants (**Supplementary Table S4**). Overall, we detected more than 1700 differentially regulated genes (DEGs; -1 ≤ log2 fold change ≥ 1, *Padj* < 0.05) in both genotypes, and 474 DEGs were commonly regulated in wild-type and knockout plants (**Figure 5A**; **Supplementary Table S5**). At first, we analyzed these 474 common DEGs and found that their expression changed in a similar way (**Figure 5B**). Among them we found the well-known pathogenesis-related genes, such as Pp3c11_1420 (the precursor of the antifungal peptide Hevein), Pp3c7_19850, Pp3c17_5160 (pathogenesis-related proteins), and Pp3c6_6560 (DIRIGENT PROTEIN). These DEGs were significantly up-regulated in wild-type and *PpRALF3* KO-infected plants (**Supplementary Table S5**). In addition, the most up-regulated common DEGs in infected plants included Pp3c3_14700 (MLRQ subunit of the NADH-ubiquinone reductase complex), Pp3c7_12870 (expansin 5-related), and Pp3c6_14470 (WRKY TRANSCRIPTION FACTOR 38-RELATED; **Supplementary Table S6**). These genes are shown to positively regulate plant immune response (Phukan et al., 2016; An et al., 2022). Most common DEGs that were down-regulated in infected plants were Pp3c14_15640 (S-TYPE ANION CHANNEL SLAH2-RELATED), Pp3c20_17620 (Asparagine synthase), Pp3c3_35020 (CCT MOTIF FAMILY PROTEIN-RELATED), and genes with unknown functions (**Supplementary Table S5**). Using the g:Profiler (Raudvere et al., 2019), we found that commonly regulated DEGs were enriched in GO terms related to response to reactive oxygen species, such as oxidoreductase activity (GO:0016491), response to reactive oxygen species (GO:0000302), and antioxidant activity (GO:0016209; **Figure 5C**; **Supplementary Table S6**). Among these genes, we found the superoxide dismutase (Pp3c17_14510), which scavenges ROS and was significantly up-regulated in both infected wild-type and knockout plants.

We next compared the GO terms of all DEGs from wild-type and knockout plants to find differences between genotypes in response to infection. In wild-type plants, the top 10 significantly enriched GO terms belonged to secondary metabolite biosynthetic processes, including polyketide metabolic process (GO:0030638), phenylpropanoid metabolic process (GO:0009698), cinnamic acid metabolic process (GO:0009803; **Figure 5D**; **Supplementary Table S6**). The upregulated DEGs belonged to these GO terms included histidine and phenylalanine ammonia-lyases (e.g., Pp3c13_9000) and chalcone synthases (e.g., Pp3c11_9040). We then used the STRING database to create association networks of DEGs from wild-type plants. Based on this analysis, the very distinct cluster of genes involved in the phenylpropanoid pathway was identified (**Figure 5E**). The transcriptional level of the genes involved in the “phenylpropanoid biosynthetic process”, “polyketide metabolic process” and “aromatic amino acid metabolic process” were significantly increased in wild-type plants in comparison to knockout line (**Figure 5F**). Among them, a group of histidine-ammonia lyases (e.g., Pp3c2_30610). These findings are in line with the previous study on the interaction between *P. patens* and *B. cinerea* (Reboledo et al., 2021). Using qRT-PCR, we additionally examined the transcription of phenylalanine ammonia-lyase (Pp3c13_9000, PAL1), pathogenesis-related protein 10 (Pp3c2_27350, PR10), and dirigent protein (Pp3c6_6545, DIR) in infected *PpRALF1* and *PpRALF2* KO lines. The induction of the PAL1 gene in the knockout genotypes was significantly lower in comparison to wild-type plants (**Supplemental Figure S7**). This is consistent with RNA-seq results on the *PpRALF3* KO genotype and implies that the phenylpropanoid pathway is suppressed in the *PpRALF* knockouts. The induction of the PR10 gene was significantly lower only in the *PpRALF2* KO line (**Supplemental Figure S7**). It should be noted that despite a similar pattern of defense gene transcription, the knockout genotypes showed different levels of *F. solani* propagation (**Figure 4D**).

Compared to wild-type plants, the GO terms of downregulated genes in *PpRALF3* KO-infected plants were mostly enriched for cell wall organization and biogenesis processes, such as pectin catabolic process (GO: 0045490), xyloglucan metabolic process (GO: 0010411), and polysaccharide metabolic process (GO: 0005976; **Figure 5G**). Based on the STRING analysis, the distinct groups of pectate lyases (e.g., Pp3c21_16990) and pectin esterases were identified in this set of DEGs (**Figure 5H**). The transcriptional level of DEGs that belonged to “pectin metabolic process”, “cell wall organization or biogenesis”, “xyloglucan:xyloglucosyl transferase activity” was decreased more pronouncedly than in wild-type plants (**Figure 5I**). This suggests a link between the role of RALF peptides in cell wall regulation and stress response. The upregulated DEGs were enriched by such GO terms as oxidoreductase activity (GO:0016491) and cell wall organization, or biogenesis (GO:0071554). However, the latter GO term included different glycosyl hydrolases and expansins. In conclusion, transcriptomic changes in wild-type plants are in line with previous data on *P. patens* response to phytopathogens (Reboledo et al., 2021) and alter such processes as ROS production and detoxification, biosynthesis of secondary metabolites with different roles in defense, and some others. The remarkable differences between *PpRALF3* KO and wild-type transcriptomes were related to genes involved in cell wall modification and biogenesis processes. Considering the known role of RALF peptides in cell wall remodeling, it can be suggested that these changes during infection result from knockout of the *PpRALF3* gene.

## 4 Discussion

RAPID ALKALINIZATION FACTOR (RALF) peptides are ubiquitous for land plants, including *Physcomitrium patens* and *Selaginélla moellendorffii*, but were not identified in chlorophyte species (Campbell and Turner, 2017). In angiosperms, RALFs are shown to modulate responses to biotic and abiotic stresses (Blackburn et al., 2020), but it is currently unknown if ancient RALF peptides helped plants to cope with different stress factors or if the immune-related RALFs appeared later as a result of tandem duplication and diversification (Cao and Shi, 2012). Here, we investigated the role of three PpRALF peptides from the model bryophyte *P. patens*, named as PpRALF1 (Pp3c3_15280), PpRALF2 (Pp3c6_7200) and PpRALF3 (Pp3c25_4180) in the immune response. Previously, PpRALF1 and PpRALF2 have been shown to promote protonema tip growth and elongation (Ginanjar et al., 2022). Although double knockouts were smaller than single knockouts, the phenotype of the mutant lines obtained was similar to those from Ginanjar et al. (2022). Herewith, *PpRALF1,3* KO plants were not observed in the previous study (Ginanjar et al., 2022) and we cannot compare these phenotypes. To explain the differences between single- and double-knockout RALF phenotypes, however, additional research is required.

According to our findings, the knockout of all three *PpRALF* genes led to consistent changes in plant proteomes, suggesting that all three PpRALFs are functional. For example, a predicted metalloprotease Pp3c15_830 was significantly downregulated in all *PpRALF* knockout lines. Plant metalloproteases are shown to be involved in growth, development, and immunity (Flinn, 2008; Mishra et al., 2021).

The role of RALF peptides in stress response is mainly explored in the context of Arabidopsis biology. In Arabidopsis, several RALF peptides have shown the ability to modulate elf18-induced ROS production (Abarca et al., 2021). Based on the amino acid compositions, we clustered PpRALFs with AtRALFs, such as AtRALF23 and AtRALF33, that negatively regulate immune response and inhibit elf18-induced ROS production in Arabidopsis (Stegmann et al., 2017; Abarca et al., 2021). However, the analysis of amino acid composition was not sufficient to distinguish PpRALF3 from PpRALF1 and 2.

In addition, the AtRALF23 and AtRALF33 were shown to interfere PAMP-triggered immunity (PTI) by binding to receptor FER-LLG1 complex and inhibition of its scaffold function (Shen et al., 2017; Rzemieniewski and Stegmann, 2022). AtRALF23 overexpression increases susceptibility to *Pseudomonas syringae* pv. *tomato* DC3000 and to the fungus *Plectosphaerella cucumerina* (Stegmann et al., 2017) and LRX3/4/5-RALF22/23-FER module negatively regulates the levels of jasmonic acid (JA), salicylic acid (SA) and abscisic acid (ABA) in Arabidopsis (Zhao et al., 2021). The hallmark of AtRALFs, that negatively regulate immune response, is the dibasic site “RR” for subtilase S1P (Srivastava et al., 2009; Stegmann et al., 2017; Abarca et al., 2021). However, there are some exceptions from this rule in Arabidopsis. In *P. patens*, only PpRALF1 protein precursor contains the corresponding “RRLL” motif (Ginanjar et al., 2022). According to our data, the PpRALF1 peptide has no role in stress response, but the knockouts of two *PpRALFs* (2 and 3) resulted in increasing resistance to bacterial and fungal pathogens - P*. carotovorum* and *F. solani*, suggesting the negative regulation of immune response by these peptides. These PpRALFs are also placed in a distinct group on the phylogenetic tree (Ginanjar et al., 2022). Our phylogenetic analysis clearly indicates that PpRALF3 diverged from PpRALF1 and PpRALF2 (**Figure 1C**).

In land plants, the immune response resulted in extensive transcriptome reprogramming (Bjornson et al., 2021; Campos et al., 2021; Reboledo et al., 2021). The early transcriptome changes include upregulation of genes involved in response to chitin and wounding at 5 minutes and participated in response to hydrogen peroxide and cell wall modification at 180 minutes after treatment by known elicitors in the model plant Arabidopsis (Bjornson et al., 2021). In our study, we found that transcriptome reprogramming in wild-type *P. patens* plants at 7 dpi affected genes that participate in response to reactive oxygen species (GO:0000302), polyketide metabolic process (GO:0030638), phenylpropanoid metabolic process (GO:0009698), cinnamic acid metabolic process (GO:0009803). The moss *P. patens* reacts to the fungal pathogen B. cinerea by reinforcing the cell wall, upregulating genes involved in the defense response, and activating the shikimate and phenylpropanoid pathways (Ponce De León et al., 2012; Castro et al., 2016; Reboledo et al., 2021). The *Marchantia* response to oomycete infection is also based on the phenylpropanoid-mediated biochemical defenses that suggest this mechanism as a hallmark of an ancestral pathogen deterrence strategy (Carella et al., 2019).

Even though the difference in the pathogenic agent - *B. cinerea* (necrotrophic fungus) vs *F. solani* (facultative parasite) and time after inoculation when samples were collected - 24-h vs 7 dpi, about 40% of DEGs in wild-type plants from our data were identical to the Reboledo dataset (Reboledo et al., 2021). However, the overlap between DEGs from *PpRALF3* KO plants and the aforementioned dataset was only 27%, suggesting specific immune responses in the knockout line.

Because our ultimate goal was to understand the role of PpRALFs in stress response, we concentrated on the comparison of the transcriptomes between wild-type and *PpRALF3* knockout plants during *F. solani* infection. About 40% of *PpRALF3* KO DEGs were identical to wild-type DEGs and changed in similar manner. The corresponding GO terms belonged to oxidoreductase activity (GO:0016491), response to reactive oxygen species (GO:0000302), antioxidant activity (GO:0016209). This finding is in line with previous studies on vascular and non-vascular plants in which increased expression of oxidative stress related genes encoding peroxiredoxins, thioredoxins, ferredoxins during infection was detected (Ponce de León, 2011; Bjornson et al., 2021; Reboledo et al., 2021). According to our results, the *PpRALF3* KO DEGs were not enriched in phenylpropanoid and cinnamic acid metabolic processes as we observed in wild-type plants, but some genes involved in the flavonoid biosynthetic process, such as chalcone synthases, were upregulated in mutant lines. Importantly, the *PpRALF2* KO and *PpRALF3* KO lines were less infected by *F. solani* than wild-type plants and *PpRALF1* KO genotype suggest their increased resistance. Therefore, the transcriptome differences between wild-type and *PpRALF3* KO plants might reflect reduced propagation of *F. solani* in knockout lines. In contrast to wild-type plants, a group of genes involved in the pectin catabolic process, such as pectate lyases and pectin esterases, were significantly downregulated in the *PpRALF3* KO plants under infection. Pectins are structural heteropolysaccharides and major components of the plant primary cell wall involved in maintaining plant growth and development, morphogenesis, defense responses, etc. (Hongo et al., 2012; Leng et al., 2017). The pectin-degrading enzymes cause plant tissue maceration, cell lysis and modification of the cell wall structure (Atanasova et al., 2018). Our findings is corroborate with the previous findings that salt stress resulted in downregulation of genes involved in cell wall organization and modification processes, such as pectin lyase-like superfamily proteins, expansins, xyloglucan hydrolases in *lrx345* (lrx3, 4, 5 triple mutants) Arabidopsis plants (Zhao et al., 2021). LRX8-LRX11 proteins are shown to interact with RALF4/19 and regulate pollen germination and pollen tube growth in Arabidopsis (Mecchia et al., 2017). The downregulation of similar cell wall related genes in our *PpRALF3* knockout line under stress conditions suggest the role of RALF peptides and the cognate receptors, such as FERONIA or LRXs, as modules that integrate plant growth and stress tolerance regulation in land plants.

In addition, it has been shown that pectin methylesterification is a subject of regulation during response to phytopathogens and a high level of pectin methylesterification correlated with an increased resistance to pathogens (Wydra and Beri, 2006; Lionetti et al., 2012). In Arabidopsis, downregulation of some pectin methylesterases during *B. cinerea* infection probably represent a defense mechanism to limit pectin demethylesterification and its subsequent degradation by fungal enzymes (Lionetti et al., 2017). Together with our results on the increased cell wall regeneration in *PpRALF2* and *PpRALF3* KO protoplasts relative to wild-type cells, it may point to changes in cell wall composition as an important factor of increased resistance to phytopathogens in the corresponding knockout lines. There is no direct evidence how different RALF peptides influence cell wall composition in plants, but knockout of some LRXs in Arabidopsis resulted in the increase of mannose and lignin if compared with wild-type plants (Draeger et al., 2015). Taken together, our findings suggest the role of PpRALF2 and PpRALF3 peptides in negative regulation of *P. patens* immune response.

We observed that *PpRALF3* and both double KO mutant lines were more tolerant to growth inhibition under salt and oxidative stress conditions. Previously, the role of AtRALF22 or AtRALF23 and their cognate receptors in coordinated regulation of cell wall integrity, growth and salt stress response was demonstrated in Arabidopsis (Zhao et al., 2018; Zhao et al., 2021). The overexpression of AtRALF22 or AtRALF23 are shown to increase sensitivity to salt stress (Zhao et al., 2018). Moreover, LRX3/4/5-RALF22/23-FER module is shown to regulate hormonal homeostasis and ROS accumulation in Arabidopsis. Thus, our findings are in line with previous studies on flowering plants and show that RALF peptides in the non-vascular plants can also participate in abiotic stress response, modulating plant growth in such conditions. The detailed mechanisms through which ancient RALF and their cognate receptors coordinated plant growth, cell wall integrity and response to distinct environmental changes (e.g., pathogen invasion) is unknown. However, the future study on genetically redundant plants, such as *P. patens*, can help elucidate the evolution and exact mechanisms of RALF peptides signaling.

## Data availability statement

The nucleotide sequence reported in this paper has been submitted to NCBI Sequence Reads Archive (SRA) with accession number PRJNA879762. The mass spectrometry proteomics data have been deposited to the ProteomeXchange Consortium via the PRIDE (Perez-Riverol et al., 2022) partner repository with the dataset identifier PXD037111.

## Author contributions

AM, IL, IF wrote the manuscript. AM, IL, NG conducted all experiments and contributed to data analysis. IF performed data analysis and supervised the study. AK, VL and DK conducted generation of knockout moss lines, also AK contributed to growth and morphological experiments. TM, EC, SE performed pathogens inoculation and moss treatment. VV, KK, TG and VB performed RNA-seq analysis. All authors contributed to the article and approved the submitted version.
