# Supplementary material for: RALF peptides modulate immune response in the moss Physcomitrium patens
Source: Front Plant Sci. 2023 Jan 27;14:1077301. doi: 10.3389/fpls.2023.1077301 (PMC9933782; doi:10.3389/fpls.2023.1077301)
Supplement: Supplementary file 1 [file DataSheet_1.pdf]

A

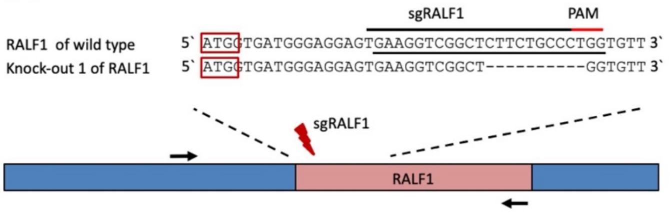

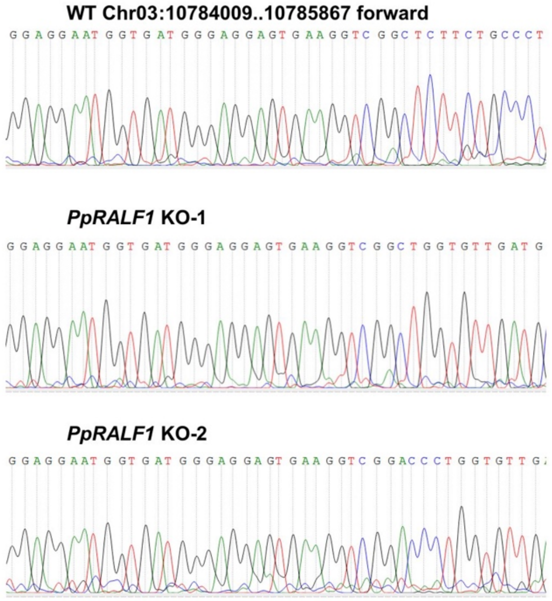

Wild type of RALF1 - MVMGGVKVGSSALVLMLAMLAFVLVQCVDRADAGHG SPIMELATTSSGLPEGFDDLRMYEDPEDQFLDEPARRLLAARRRSYISYGALNRN RSPCPARSGRSYYTPNCNSNAGPARPYTRGCLRITRCQRV*

Knock-out 1 of RALF1 - **MVMGGVKVG**WC*

Knock-out 2 of RALF1 - **MVMGGVKVG**PWC*

B

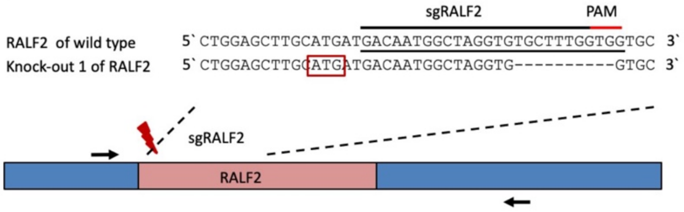

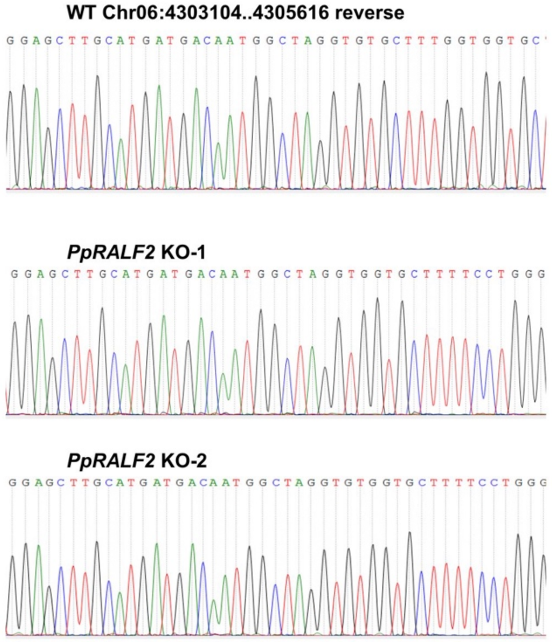

Wild type of RALF2 - MMTMARCALVVLFLGCLVLEAQAQAPFAAVAPSPFTGFPPQEAPAFAGSGGPVL YITYGALRANRSPCPAGAGRSYYTPNCGAASGPPNPYSRGCSYITRCARV*

Knock-out 1 of RALF2 - MMTMARWCFSWVVWSWKPRRRLRSLP*

Knock-out 2 of RALF2 – MMTMARCGAFPGLFGPGSPGAGSVRCRSSESLHRV PTAGGSRFRGLWWSGSLHHLRCSKSKPQSMPCWSRTKLLHAQLRCCIGTPEPI QQGLQLHHPLCSRLRRCTWVLPRSEMHMPSSTTMVE*

C

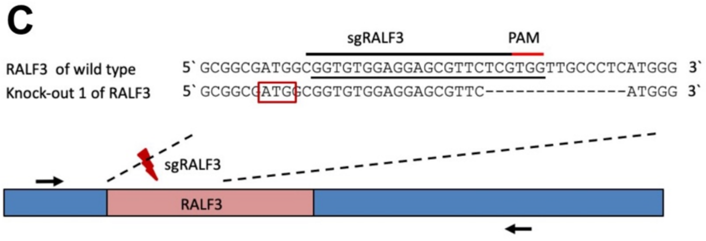

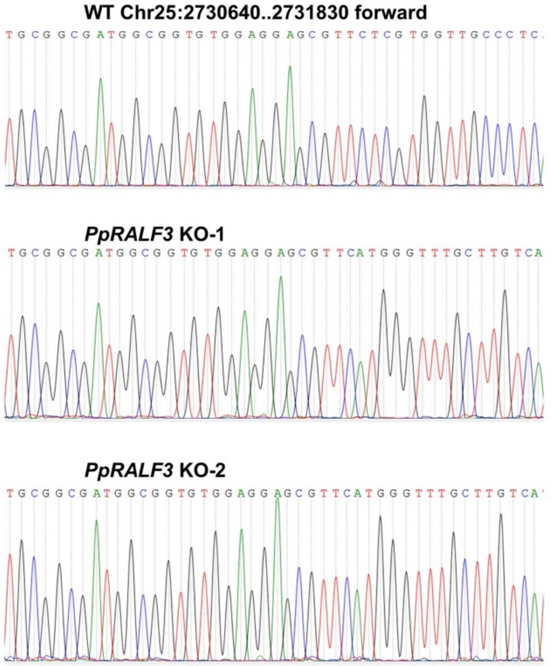

Wild type of RALF3 -
MAVWRSVLVVALMGLLVMGIQAEQQLGFSPGAIYPDAHRVLAAAPNYYISYGSL
NADRAPCPASSSGRSYYTTNCQSSGTPQPYVRSCSQITRCARG*
Knock-out 1 of RALF3 - MAVWRSVHGFACHGYPS*
Knock-out 2 of RALF3 - MAVWRSVHGFACHGYPS*

D

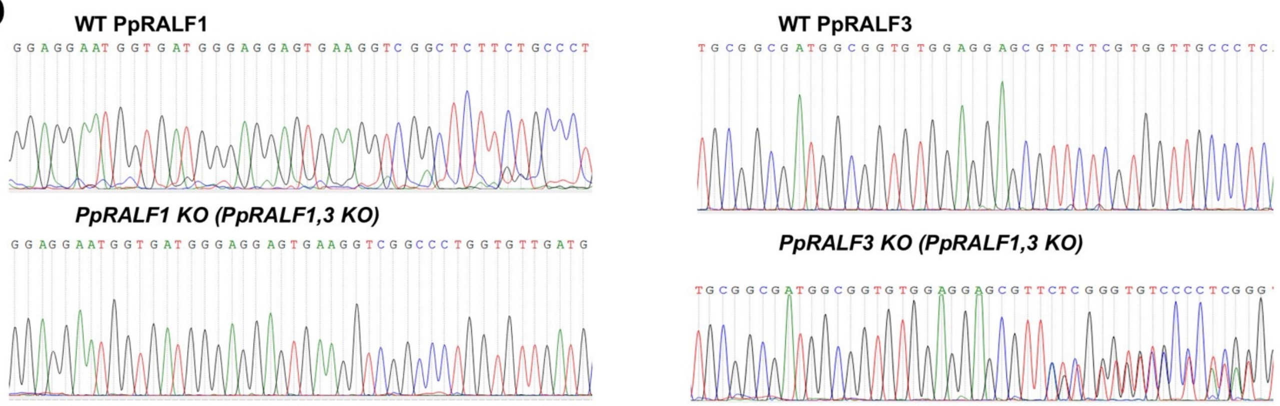

Knock-out of RALF1 in PpRALF 1,3 KO - MVMGGVKVGPGVDAGNAGIRAGAMRGQGGRGSWKPHHGTRYHVLRSPRGVR*
Knock-out of RALF3 in PpRALF 1,3 KO - MAVWRSVLGCPLGFARVGDTHSEPPCVFPPCAFYPYAPRVCGAAPI*

E

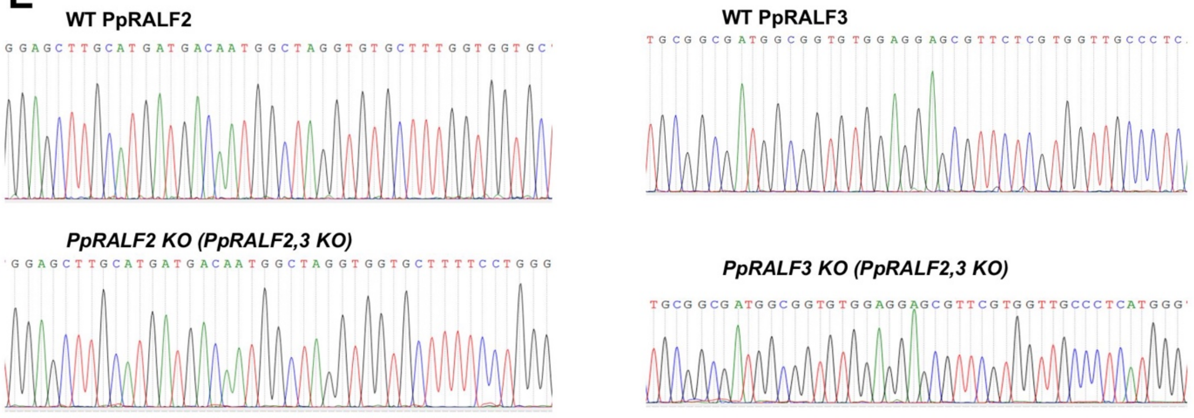

Knock-out of RALF2 in PpRALF 2,3 KO - MMTMARWCFSWVVWSWKPRRRLRSLP*
Knock-out of RALF3 in PpRALF 2,3 KO - MAVWRSVRGCPHGFACHGYPS*

Supplementary Figure S1. A – The schematic representation of sgRNAs designed to knockout of PpRALF1 peptide, the results of Sanger sequencing of obtained mutant lines and deduced amino acid sequences; **B –** The schematic representation of sgRNAs designed to knockout of PpRALF2 peptide, the results of Sanger sequencing of obtained mutant lines and deduced amino acid sequences; **C –** The schematic representation of sgRNAs designed to knockout of PpRALF3 peptide, the results of Sanger sequencing of obtained mutant lines and deduced amino acid sequences. *Black arrows* indicate the positions of primers for PCR analysis. *Red* frames indicate the positions of start codon; **D** - The results of sequencing of RALF genes in obtained mutant lines and deduced amino acid sequences for PpRALF1,3 double knockout; **E** - The results of sequencing of RALF genes in obtained mutant lines and deduced amino acid sequences for PpRALF2,3 double knockout

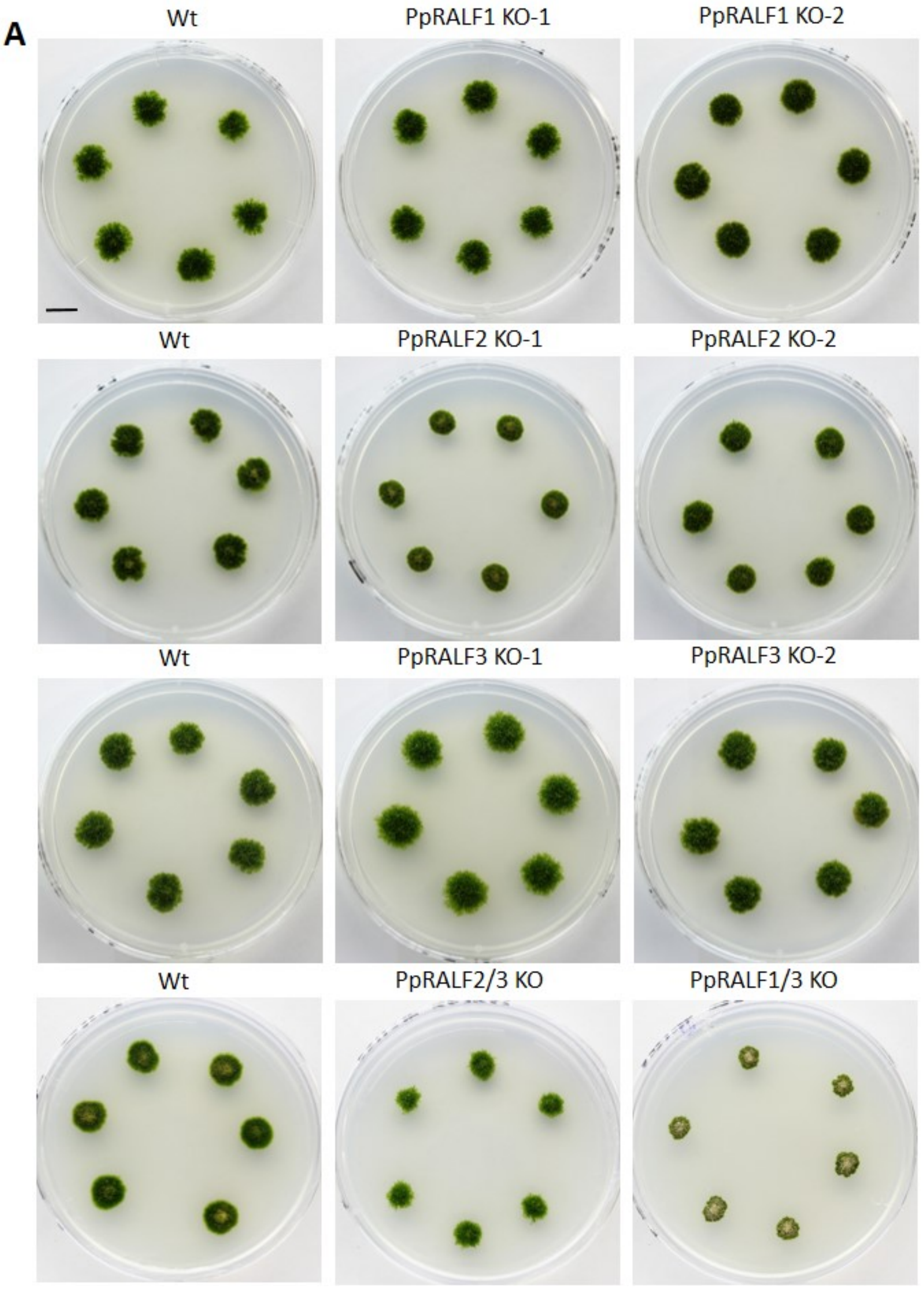
A
Wt
PpRALF1 KO-1
PpRALF1 KO-2
Wt
PpRALF2 KO-1
PpRALF2 KO-2
Wt
PpRALF3 KO-1
PpRALF3 KO-2
Wt
PpRALF2/3 KO
PpRALF1/3 KO

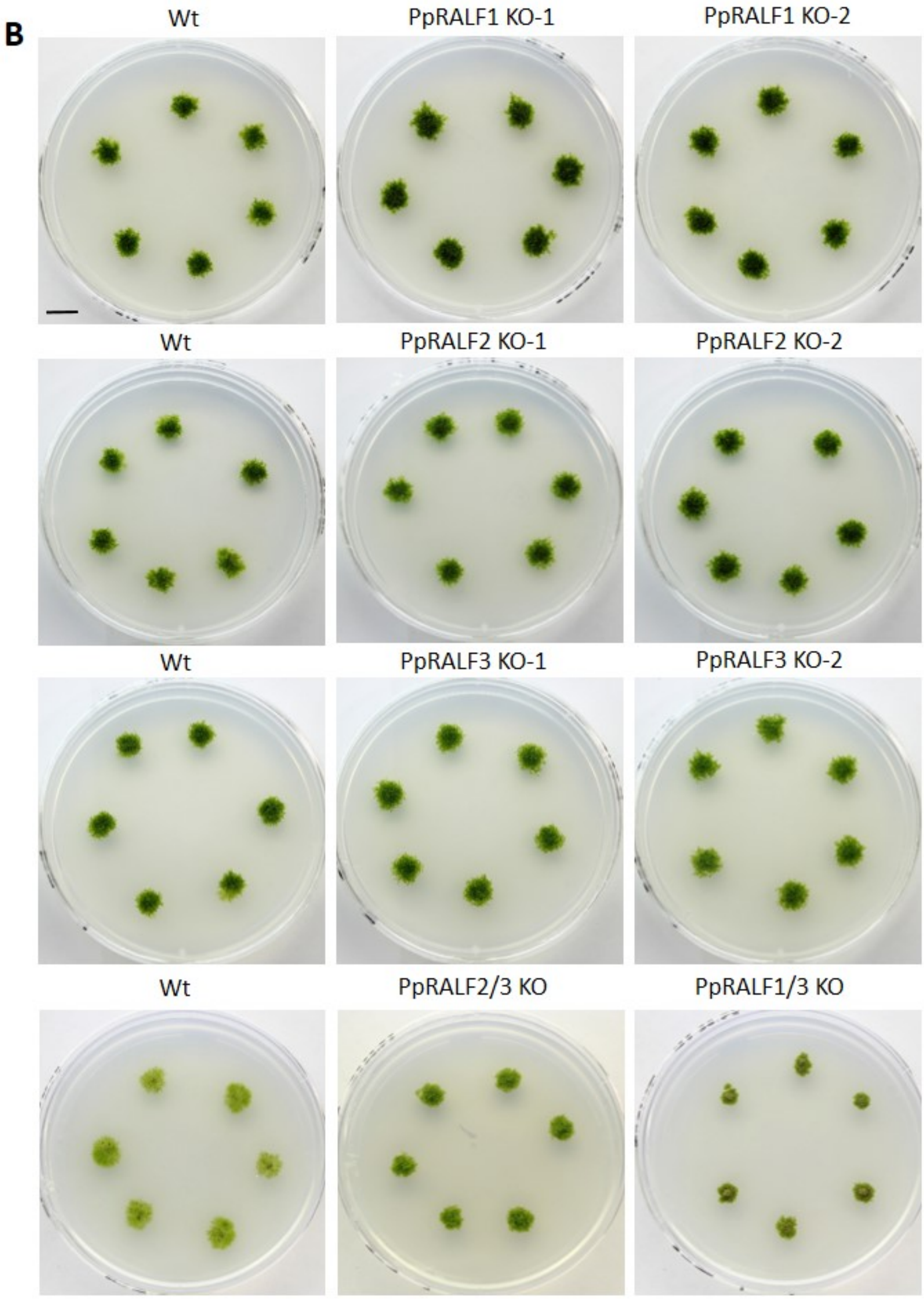

Supplementary Figure S2. Protonemata of wild type and knockout lines grown on **A** – BCDAT medium and **B** - BCD medium. Scale bar: 10 mm

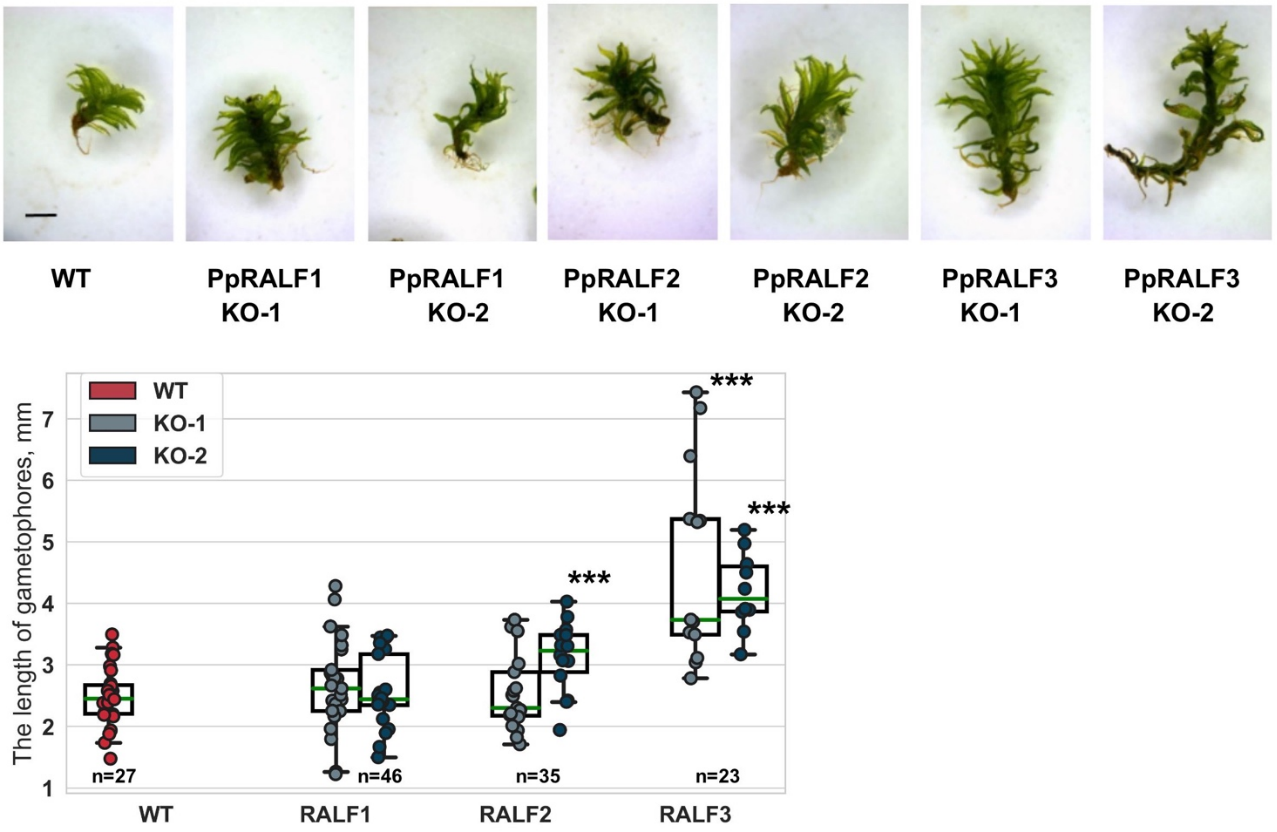

Supplementary Figure S3. The length of gametophores of wild-type and knockout plants. Analysis of variance and Tukey's HSD post hoc tests were performed ****P* < 0.001

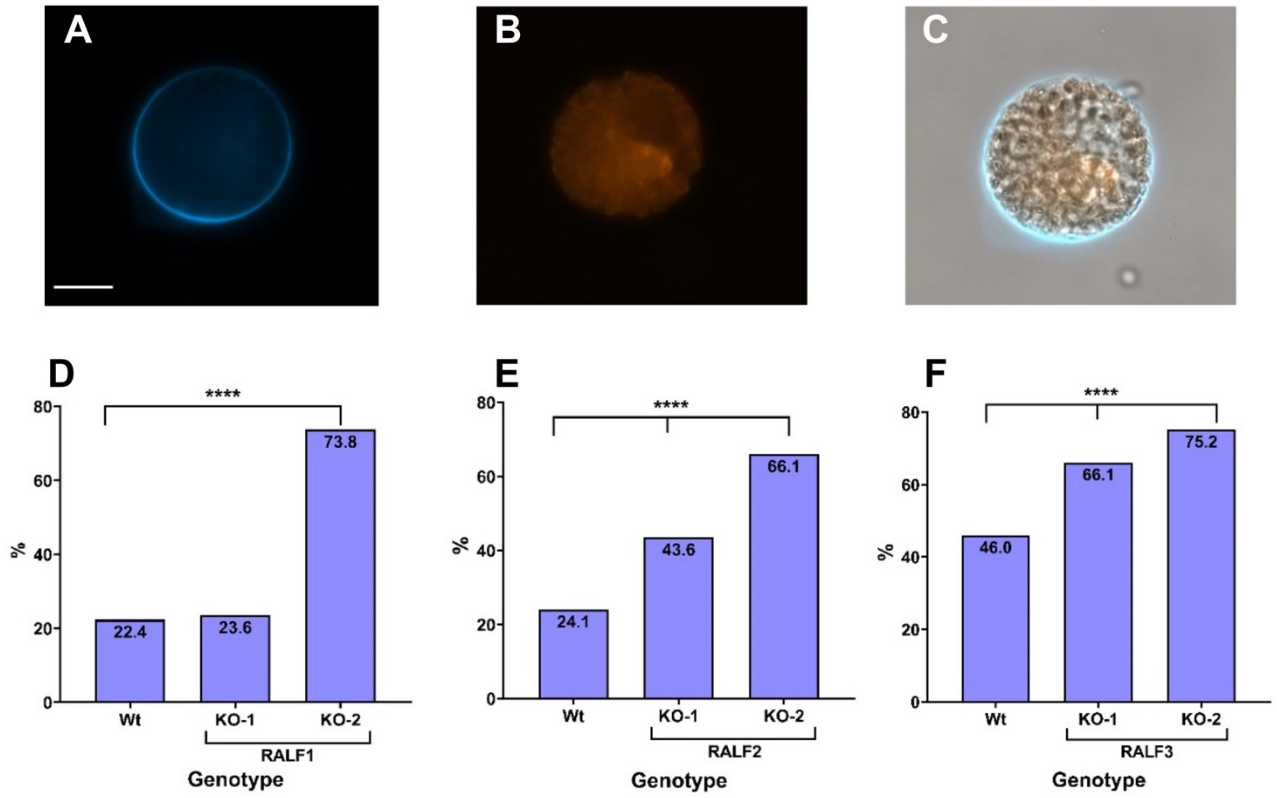

Supplementary Figure S4. **A** – Protoplast cell stained with Calcofluor White; **B** – Chloroplast autofluorescence in protoplast cell; **C** – combined A and B picture; **D** – The percent of regenerated protoplast in protoplast from PpRALF1 genotype in comparison to wild-type plants; **E** – The percent of regenerated protoplast in protoplast from PpRALF2 genotype in comparison to wild-type plants; **F** – The percent of regenerated protoplast in protoplast from PpRALF3 genotype in comparison to wild-type plants. Analysis of variance and Tukey's HSD post hoc tests were performed ****$P < 0.001$

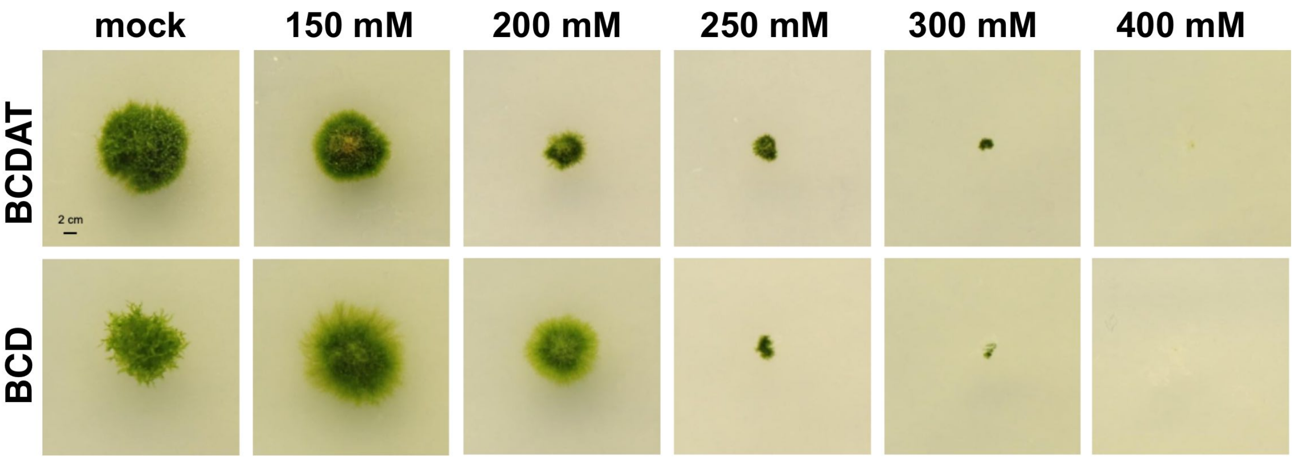

Supplementary Figure S5. The effect of different NaCl concentrations on growth rate in wild-type plants on two types of growing media: BCD and BCDAT (BCD medium supplemented with ammonium tartrate)

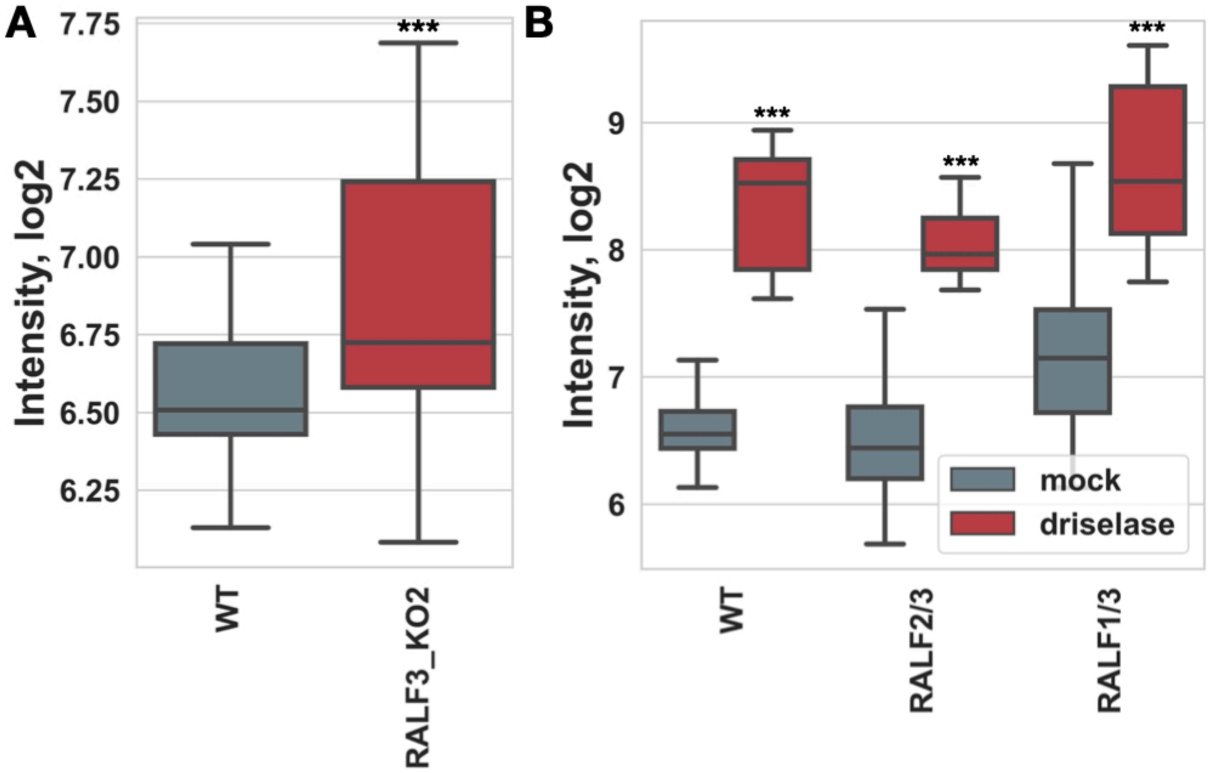

Supplementary Figure S6. Effect of PpRALF peptide knockouts on ROS production in wild-type and **A** - *PpRALF3* KO-2 line or **B** - double knockout lines treated with 0.0025% driselase. Analysis of variance and Tukey's HSD post hoc tests were performed. ***$P < 0.001$

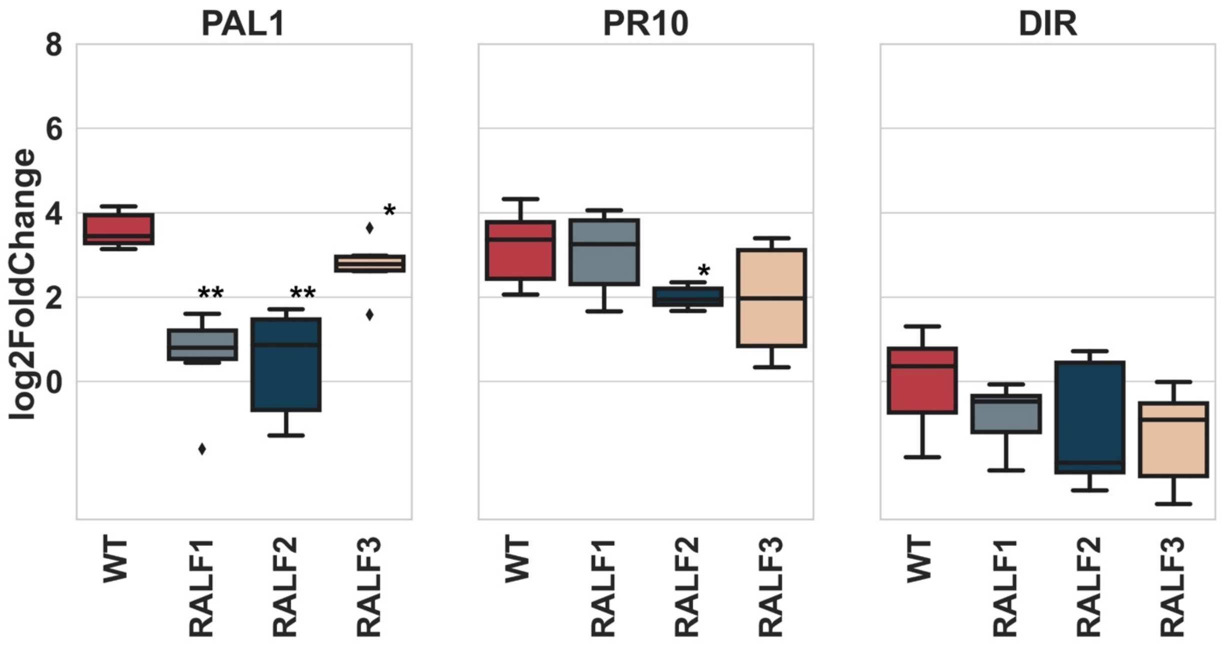

Supplementary Figure S7. The results of quantitative reverse transcription PCR (RT-qPCR) analysis of the transcriptional level of phenylalanine ammonia-lyase (PAL1), pathogenesis-related protein 10 (PR10), dirigent protein (DIR) genes after *F. solani* infection. Mann-Whitney U tests were performed. $^{**}P < 0.01$, $^{*}P < 0.05$. Log2FoldChange is logarithmic 2-ΔΔCT values
